# Supplementary material for: A Highly Homogeneous Airborne Fungal Community around a Copper Open Pit Mine Reveals the Poor Contribution Made by the Local Aerosolization of Particles
Source: Microorganisms. 2024 May 4;12(5):934. doi: 10.3390/microorganisms12050934 (PMC11123957; doi:10.3390/microorganisms12050934)
Supplement: Supplementary file 1 [file microorganisms-12-00934-s001.zip › Fuentes_et.al_Fig_S1.pdf]

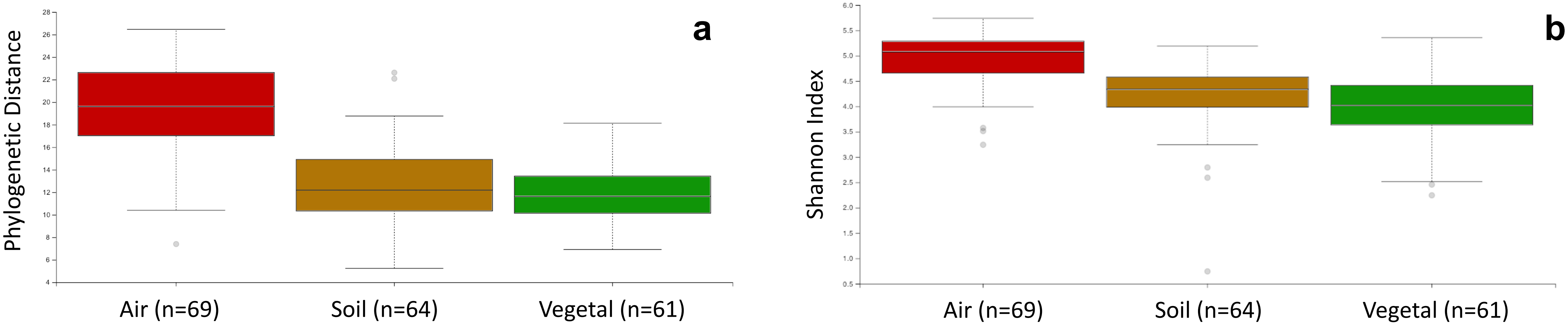

Figure S1. Alpha-diversity of the three communities: air (red), soil (brown) and vegetal detritus (green). The median is depicted in the center of each box. Similar to richness in Figure 2, Faith's phylogenetic diversity (**a**) and Shannon index (**b**) yielded similar results: air is more diverse than soil and vegetal debris.
