# Supplementary material for: A Highly Homogeneous Airborne Fungal Community around a Copper Open Pit Mine Reveals the Poor Contribution Made by the Local Aerosolization of Particles
Source: Microorganisms. 2024 May 4;12(5):934. doi: 10.3390/microorganisms12050934 (PMC11123957; doi:10.3390/microorganisms12050934)
Supplement: Supplementary file 1 [file microorganisms-12-00934-s001.zip › Fuentes_et.al_Fig_S3.pdf]

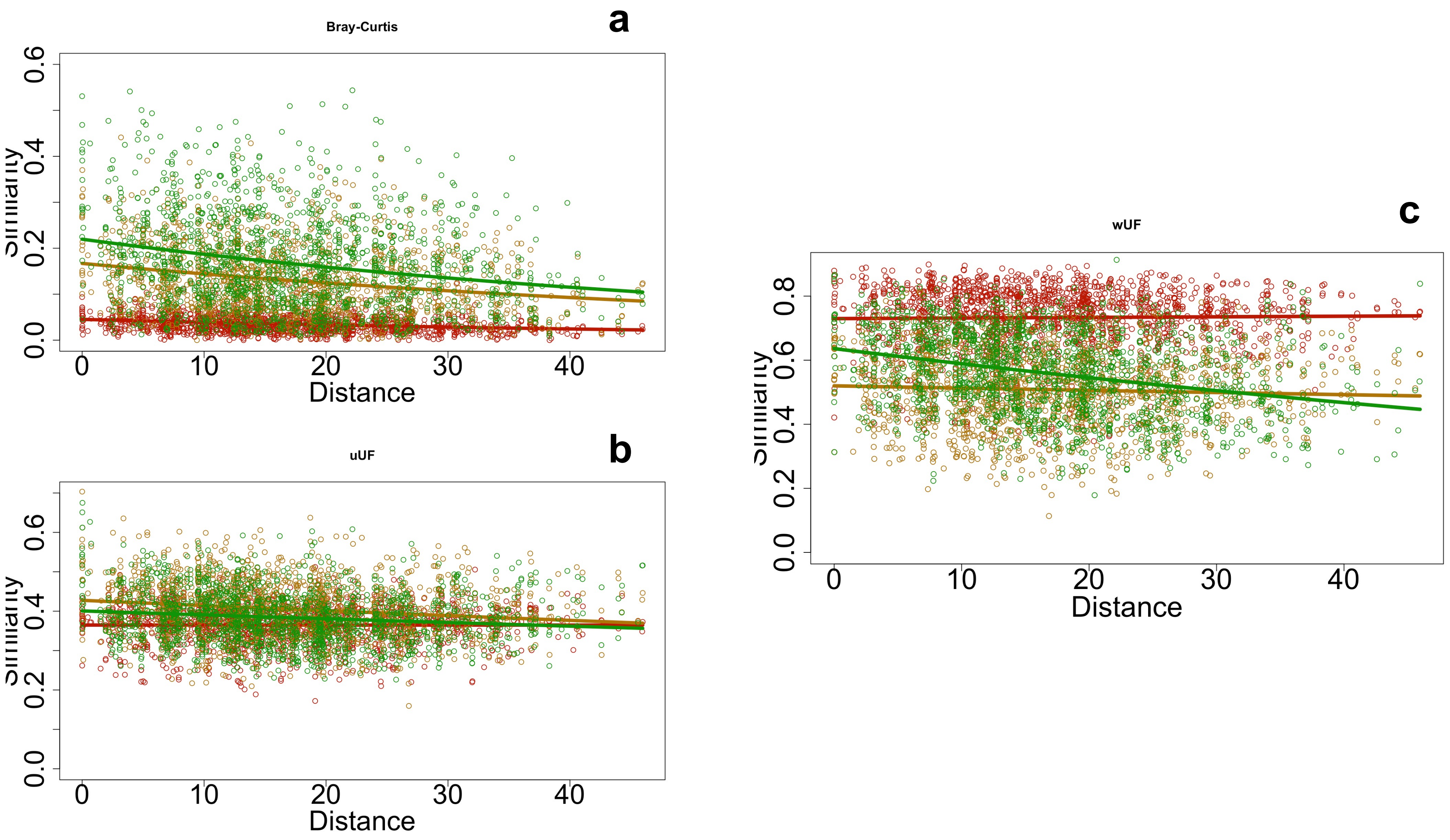

Figure S3. Similarity distance-decay plots of the three communities: air (red), soil (brown) and vegetal detritus (green). **a**) Bray-Curtis dissimilarity, **b**) Unweighted UniFrac, **c**) Weighted UniFrac.
